# The Introduction of Allochthonous Olive Variety and Super High-Density System in the Abruzzo Region: A Study on Olive Oil Quality

**DOI:** 10.3390/foods12061292

**Published:** 2023-03-17

**Authors:** Federica Flamminii, Sara Gaggiotti, Alessandro Chiaudani, Dario Compagnone, Angelo Cichelli

**Affiliations:** 1 Department of Innovative Technologies in Medicine and Dentistry, University “G. d’Annunzio” of Chieti-Pescara, Via dei Vestini, 66100 Chieti, Italy; 2 Faculty of Bioscience and Technology for Food, Agriculture and Environment, University of Teramo, Via R. Balzarini 1, 64100 Teramo, Italy

**Keywords:** super-high density, olive oil quality, Lecciana, Koroneiki, Frantene

## Abstract

The transition to a sustainable economic and environmental management of olive oil sector needs to be implemented in both national and regional territories through the introduction and development of innovative growing systems and variety. In this study, the olive oil quality parameters of local and allochthonous varieties cultivated in different orchards located in the Abruzzo region (Italy), using traditional and super high-density systems, were analyzed. Frantene, Lecciana, Koroneiki, and a mix of Arbequina and Lecciana provided olive oils rich in flavonoids and secoiridoids compounds with respect to the local varieties Frantoio, Leccino, and a mix of Dritta, Leccino, and Pendolino. Oleic/linoleic ratio was influenced by cultivar and training systems with super high-density olive oils rich in oleic acid. Frantene showed a peculiar fatty acid profile different from cultivars grown in the same location; moreover, interesting similarities were found between Frantene and the mix of Dritta, Leccino, and Pendolino in terms of health-related compounds. The potential development of innovative sustainable training system to improve olive oil quality was highlighted. The study’s results identify olive varieties suitable for super high-density systems spread in the Abruzzo region, representing a valid alternative for the olive growers to improve both the quality of the olive oil, as well as the company’s income.

## 1. Introduction

The olive oil sector represents a fundamental pillar in the economy and landscape for both Italy and the Abruzzo region, located in the center of the peninsula. The Italian olive oil sector is characterized by limited cultivated areas, different farming systems, extreme fragmentation of the production structures and a vast national olive germplasm that increases the variability of the final product [1]. These general national aspects reflect the regional situation and represent the main drawback for the development of the olive oil sector. Furthermore, the scarce income generated does not allow farmers to introduce all the innovations necessary to be competitive on the international market with other more productive regions and nations. 

Concerning the Abruzzo region, even if the cultivation finds the maximum production along the coastal hill, olive trees are widely distributed overall the regional territory, starting from the sea to the foothills of Majella and Gran Sasso at 600–700 m above sea level thanks to local varieties that are particularly suitable for these environments [2]. Although the high quality of the regional production is highlighted, confirmed by few scientific research studies regarding autochthonous olive cultivars of the Abruzzo region [2,3,4], no significant economies are realized due to the presence of technical–structural, organizational and commercial problems along the entire supply chain. 

Generally, in the Abruzzo region, most olive orchards are exploited under traditional plantation systems, characterized by a reduced number of trees per hectare, being rainfed and poorly mechanized. The introduction of structural innovation, technology and techniques, through the adoption of intensive planting scheme with high-density (HD; 250–700 trees ha^−1^) and super high-density (SHD; 1500 trees ha^−1^) systems, could represent an important strategy for the local olive oil sector. Such intensive systems, characterized by increased yields production, earlier bearing, efficient harvesting, higher financial return, reduced production costs and environmental impact per unit of product [1], require considerable technical skill to manage these orchards successfully [5]. 

This new cultivation strategy has driven a revolution in olive oil production throughout the introduction of olive cultivars particularly designated for HD and SHD systems that can overcome difficulties in production; several trials evaluated the behavior of local and foreign cultivars with high planting densities from around 800 to almost 2600 trees ha^−1^, in particular, Arbequina, Arbosana, Koroneiki and Sikitita, which are the most common intensively planted varieties around the world [6,7,8]. In the Umbria region (Italy), the adaptability to the SHD olive training system and to mechanical harvesting of four Italian cultivars, Frantoio, Leccino, Maurino and Moraiolo, was compared with Arbequina; the growth habit fitted well for the high-density systems for all the autochthonous varieties, and Maurino seemed to be suitable for super intensive oliviculture in terms of vegetative growth and reproductive aptitude [7]. A similar study was assessed for Arbequina, Arbosana, Don Carlo© and FS 17© cultivated in the Abruzzo region in high-intensive olive orchards. The results revealed that the most productive cultivars were Arbosana and Arbequina, with the highest efficiency index (yield/m^3^ of crown), while FS 17© and Don Carlo© had a lower production, respectively. However, from a chemical and sensory point of view, Don Carlo© and FS 17© oils had a higher phenolic content and resulted in being more bitter and spicier than Arbequina and Arbosana [8]. A study on agronomic and qualitative characteristics of olive genotypes selected in central Italy revealed that the crossbreeding of Dritta and Gentile di Chieti reduced the vigor of parent’s genotypes, resulting in being suitable for planting at high density [9]. Moreover, in 1998, from a controlled cross between cv. Arbosana and cv. Leccino, Lecciana© was developed, which is a new olive cultivar suitable for SHD that reflects all the vegetative and productive traits required for efficient, sustainable olive growing intensification [10]. It is necessary to highlight that despite growing system intensification increases agrochemicals use, irrigation and mechanization, with a consequent greater environmental impact [1], the super-intensive olive groves could be renamed super-sustainable olive groves since they reduce the olive carbon [11] and water footprint (WF) as well [11,12]; consequently, these new growing techniques should be considered as a powerful marketing tool for olive oil companies to orient the decision making of consumers increasingly attentive to social-environmental and food quality issues [1].

The aim of this research was to analyze the olive oil quality parameters of allochthonous olive cultivars grown in super high-density orchards and traditionally trained varieties, cultivated in the Abruzzo region; Lecciana, Frantene, Koroneiki and a mix of Lecciana and Arbosana were studied and compared with local cultivar as Frantoio, Leccino and the blend Dritta, Leccino and Pendolino. Legal indices and quality-functional parameters such as tocopherols, phenolic compounds and fatty acids profile were evaluated and similarities among samples were also assessed by cluster analysis. The results of this study identify olive varieties suitable for super high-density systems cultivated in the Abruzzo region and represent a valid alternative for the olive growers to improve both the quality of the olive oil, as well as the company’s income, in regional as well as in national areas.

## 2. Materials and Methods

### 2.1. Plant Material and Oils

**Figure 1:**
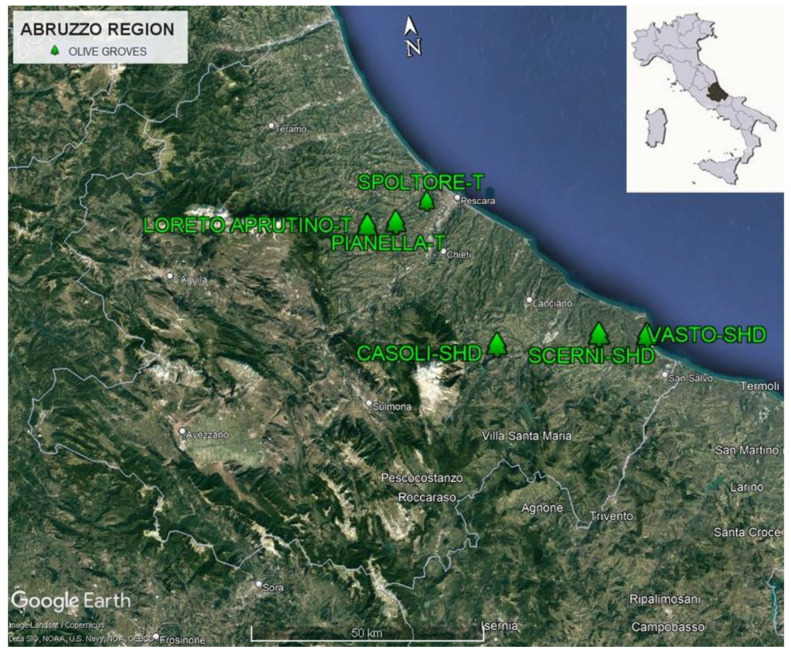
Location of the olive groves in Abruzzo region.

Plant materials collection and oils extraction were conducted during the harvesting years 2020 and 2021. Different olive cultivars, belonging to Pescara and Chieti provinces of Abruzzo region, were identified and selected (Figure 1). Frantoio and a mix of Leccino, Dritta and Pendolino cultivated, respectively, in Loreto Aprutino (42°24′07.4″ N 13°58′04.4″ E), Spoltore (42°25′33.7″ N 14°06′47.6″ E) and Pianella (42°23′41.3″ N 14°02′36.6″ E) municipalities, were selected as benchmark cultivars of POD “Aprutino Pescarese” varieties, cultivated with a traditional training system spaced 6 × 6 m^2^; Frantene, Lecciana, Koroneiki and a mix of Arbosana and Lecciana were selected from more southern SHD orchards (4 × 1.4 m^2^), located in the municipalities of Vasto (42°04′21.2″ N 14°44′20.3″ E), Casoli (42°08′39.4″ N 14°18′06.6″ E) and Scerni (42°06′19.9″ N 14°34′53.5″ E), respectively; Frantene trees were provided and planted by Agromillora Catalana Sa (Sant Sadurni d’Anoia, Catalunya, Spain) in Vasto (42°04′21.2″ N 14°44′20.3″ E).

Olives were mechanically harvested in October, in non-experimental orchards, from 20 plant samples for each cultivar; fruits were at a medium level ripening, specific for each variety and defined from each producer. Traditional and SHD were rain fed and irrigated, respectively. All the olive oils were obtained with industrial three phase continuous extraction systems within 12–24 h from the harvest. Oils were collected during the extraction, packaged in dark glass bottles of 750 mL and stored in dark conditions at about 18 °C until analysis. Oil samples were coded with alphanumeric numbers based on the harvest year (Table 1); “B” and “C” correspond to 2020 and 2021, respectively. The trees and industrial olive mills were the same during the two years. Climatic data were provided by the meteorological monitoring network belonging to the Regional Hydrographic Mareographic Service of Civil Protection. Average data of temperature, thermal excursion and cumulative rainfall were referred from January to October (Table 1 and Figure S1, Supplementary Material). 

### 2.2. Olive Oils Chemical Characterization

Olive oils were assessed for legal indices as free acidity (FA), peroxides value (PV), UV light absorption (K_232_, K_270_) and fatty acids, determined according to the analytical methods described in EC Regulations [13,14]. 

### 2.3. Phenol Extraction 

The extraction of phenolic compounds from oil samples was carried out as described in Del Carlo et al. [15] with slight modifications. Two grams of olive oil were weighed and vortexed for 1 min with 10 mL of hexane. Commercial solid-phase extraction (SPE) C_18_ cartridges (2 g, 6 mL) (International Sorbent Technology, Hengoed, UK) was activated with 2 × 5 mL of methanol and conditioned with 2 × 5 mL of hexane. The sample was loaded onto the cartridge and subsequently washed with 2 × 5 mL of hexane to eliminate all the lipophilic fraction. The phenolic compounds were then recovered by eluting with 2 × 5 mL of methanol. The eluate was dried by means of a rotavapor apparatus (30 °C, 150 RPM) and subsequently reconcentrated in 2 mL of methanol.

### 2.4. Total Phenolic Compounds

The Folin–Ciocalteu method was used for the determination of total polyphenols [16]. The phenolic extract was mixed with 0.5 mL of Folin–Ciocalteu reagent and 4 mL of deionized water, and the solution was allowed to react for 3 min. Subsequently, 1.5 mL of Na_2_CO_3_ was added and filled up with water up to 10 mL. The solutions were stirred at room temperature for 60 min in the dark, and the absorbance was detected spectrophotometrically at 725 nm. The calibration curve was made using hydroxytyrosol (HTR) (Sigma-Aldrich, Milan, Italy) as standard, and results were expressed as mg L^−1^.

### 2.5. Antioxidant Activity 

Antioxidant assays (ABTS) was performed according to [17] with slight modifications. Different volumes of extract or standard were mixed with ABTS^•+^ reagent up to a final volume of 1 mL; after 5 min of reaction at room temperature in the dark, the absorbance was measured at 734 nm. Each measurement was compared with a control sample prepared without the addition of phenolic compounds. The quantity of ABTS consumed is evaluated as a percentage of decrease in absorbance and was calculated with the formula D% = ((White Abs − Sample Abs) − White Abs) × 100. The calibration curve was made using hydroxytyrosol as standard (Sigma-Aldrich, Milan, Italy).

### 2.6. Total Tocopherols

Total tocopherols were quantified according to ISO 9936:2016 [18] using an Agilent 1100 HPLC-DAD-FLD system (Agilent Technologies, Milano, Italy) with a Supelco Discovery C_18_ column (Sigma-Aldrich, St. Louis, MO, USA) 25 cm × 4.6 mm (i.d. 5 μm) set at 35 °C. The injection volume was 10 μL, and elution was conducted in isocratic mode at a flow rate of 1 mL min^−1^ with a mobile phase composed of 75% methanol/20% acetonitrile/5% Tetrahydrofuran (*v*/*v*). Detection was performed at 292 nm in UV and quantification was assessed by fluorescence detector at l_exc_ = 290 nm and l_em_ = 330 nm. Total tocopherols were quantified in comparison with a multipoint calibration curve obtained using α-tocopherol (Merck, Darmstadt, Germany) as standard. Results were expressed as mg of α-tocopherol kg^−1^ oil. 

### 2.7. Phenolic Profile

Polyphenolic profile was determined according to [19] by a Nexera XR UHPLC system (Shimadzu, Tokyo, Japan) coupled to a Qtrap 4500 mass spectrometer (Sciex, Toronto, ON, Canada) equipped with a heated ESI source (V-source). The analytes were separated using an Excel 2 C18-PFP column (10 cm × 2.1 mm ID) from ACE (Aberdeen, UK) packed with 2 μm particles and equipped with a security guard. The mobile phases were 1% acetic acid in water (A) and acetonitrile (ACN) (B). The flow rate was set at 0.300 mL min^−1^ for a total run of 15 min. The acquisition and quantification of ion currents was performed in MRM mode. Standard compounds, namely, tyrosol, hydroxytyrosol, diosmetin, luteolin, apigenin, oleacein and oleocanthal, were purchased from Sigma-Aldrich (Milan, Italy). Data collection and processing were performed with Analyst 1.6.2 software and quantification with Multiquant 3.0 software (Sciex). The selected ions, together with the main HPLC-MS/MS parameters are reported in Table S2 (Supplementary Material).

### 2.8. Statistical Analysis

Olive oil results were expressed as mean ± standard deviation of three replicates (n = 3) from each sample. Analysis of variance (one-way and two-way ANOVA) was used to assess the effect of cultivar and year on olive oil parameters (Table S1, Supplementary Material), and Tukey test established the statistical significance (0.05) among the different mean values. Additionally, datasets were also exported into ClustVis software for clustering analysis based on similarity; hierarchical dendrograms were produced using the complete agglomeration method on Euclidian distance matrices and Ward linkage. Heatmaps outputs highlighted the data matrix values in colored gradient cells based on original data. Data analyses were carried out by XLSTAT software (Addinsoft SARL, New York, NY, USA) and ClustVis, a web tool freely available at http://biit.cs.ut.ee/clustvis/ (accessed on 11 October 2022) [20].

## 3. Results and Discussion

The composition of extra virgin olive oil (EVOO) is a result of several factors such as genotypic potential, environmental factors, fruit ripening, harvest time, agricultural factors (irrigation, sunlight, orchard management) and technological factors such as the method applied for oil extraction and the storage conditions, which can vary every year; for this reason, it is not easy to individuate a precise cause effect relationship between them [21,22]. 

### 3.1. Free Acidity, Peroxide Value, K_232_, K_270_

As observed in Table 2, all the oil samples showed FA, PV and spectrophotometric indexes data below the legal limit for EVOO category according to the EU Regulations [13,14]. Between the two seasons, significant differences in FA value were highlighted for most samples (*p* < 0.05) except for Leccino, Arbequina/Lecciana (A/L) and Koroneiki with mean biennial values of 0.34%, 0.18% and 0.32%, respectively. Cultivar (*p* < 0.0001) and combination of factors as cultivar and year (*p* < 0.0001) significant influenced free acidity results (Table S1). 

About the oxidative status of olive oil, the highest value of PV was observed in sample B14 (8.0 ± 0.96), while no significant differences (*p* > 0.05) were reported for the other samples regardless the cultivar and year with values ranging from 6.0 ± 0.7 to 4.5 ± 0.5 meqO_2_ kg^−1^. Samples B10, C2, B12 and C5 registered the lowest values (4.2 ± 0.6 to 4.0 ± 0.5). As highlighted for the free acidity, the cultivar (*p* < 0.0001) and combination of factors as cultivar and year (*p* < 0.0001) significant influenced PV (Table S1). Despite the ANOVA results, which clearly delineated the influence of cultivar, year and their combination, the differences could be ascribable to biotic and abiotic factors, indeed, as reported in the literature, different free acidity and peroxides values could be associated with the activity of exogenous and endogenous olive lipase and lipoxidase, in the intact fruit cells, during fruit maturation or in the milling mass, during the process of oil extraction. The level of activity of this enzyme could depend on multiple variables such as the variety, the level of maturity, the temperature, or the humidity [23,24]. Furthermore, biotic or abiotic fruit damage could greatly influence the level of those parameters [25].

In addition to PV, K_232_ measures the primary oxidation compounds, indicating conjugated double bonds from hydroperoxides, while *K*_270_ is an indicator of secondary oxidation products generated during the breakdown of hydroperoxides. The K_232_ value ranges between 1.72 and 1.23, while the K_270_ value ranges between 0.12 and 0.04. The former is influenced by cultivar (*p* < 0.05) and year (*p* < 0.0001), while the latter is also susceptible to the interaction between the cultivar and harvest year (*p* < 0.0001) (Table S1). 

### 3.2. Quality and Functional Parameters: Total Phenolic Content, Antioxidant Activity and Total Tocopherol

The uniqueness of extra virgin olive oil is due to the abundance of bioactive compounds such as hydrophilic phenols, phytosterols, tocopherols, carotenes and fatty acids, mainly PUFA and MUFA, that provide several functional properties as well as a long product storage time due to their high oxidative stability [21]. The results of TPC, TT and antioxidant activity are presented in Table 3. 

The highest content of TPC was observed in Frantene (C13) and Koroneiki (B13) with 450 ± 0.5 and 448 ± 05 mg HTR kg^−1^, respectively. A low content of phenolic compounds was reported for Lecciana (B12), Koroneiki (C4) and A/L (B4) with values of 136 ± 16, 154 ± 18 and 145 ± 17 mg HTR kg^−1^, respectively. Significant differences (*p* < 0.05) were reported in Frantoio, Frantene and Koroneiki varieties considering the two-harvest year.

Considering the mean values of the two years (data not shown), L/D/P and Frantene showed the highest TPC content, respectively, of 360 and 358 mg HTR kg^−1^, while A/L and Lecciana reported the lowest value of approximately 172 mg HTR kg^−1^, unlike to results observed from other authors for Lecciana cultivar with a higher value of 458 mg kg^−1^ cultivated in Puglia region [10]; intermediate values of TPC were associated with the local varieties Leccino (≈278 mg HTR kg^−1^), Frantoio (≈225 mg HTR kg^−1^), and Koroneiki of about 301 mg HTR kg^−1^, the results of which were in line with the great variability data observed in the literature for the Greek variety [26]. Based on the ANOVA results (Table S1), the cultivar and the combined effect year/cultivar significantly affected the TPC content (*p* < 0.0001). As previously reported, the great fluctuation in the phenolic content is influenced by chemical and enzymatic alterations during olive ripening [27], and can also be associated with the spacing, orientation, fruit position within the canopy, and quality of irradiance depending on the training systems adopted; in particular, the canopy upper layers with high irradiance show an increasing response of phenolic compounds [28,29], while agronomical factors such as high irrigation doses could reduce phenolic content [30,31].

With respect to health claims authorized by EFSA for olive oil [32] and in particular the “olive oil polyphenols” one, characterizing olive oils containing at least 300–350 mg kg^−1^ of phenolic compounds, both samples of blends Leccino/Dritta/Pendolino (B14, C1), Frantoio (B2), Frantene (C13) and Koroneiki (B13) can benefit from it, while the rest of the oil samples (Leccino, Lecciana and Arbequina/Lecciana) belong to the low-content category (20–200 mg kg^−1^) [33]. 

The antiradical scavenging activity of the phenolic extract assessed the ability of antioxidants to scavenge the ABTS radical cation. The results reported in Table 3 confirm the trend observed for TPC. Frantene (C13), Frantoio (B2), L/D/P (B14, C1), Leccino (B10) and Koroneiki (B13) showed higher activity, ranging from 530 ± 64 to 400 ± 48 mg HTR kg^−1^. Significant differences (*p* < 0.05) were highlighted for Frantene, Frantoio and Koroneiki between the two-harvest year. Pearson coefficient correlation was used to highlight the possible influence of hydrophilic compounds on oxidative stability of olive oils. A good correlation (0.974) was reported between mean values of TPC content and antioxidant activity of olive oil samples. 

Concerning the Tocopherol content, three isoforms are in general present in EVOO: α-, β- and γ-tocopherol. α-Tocopherol can be found in its free form and represents more than 90% of the total [21].

Results reported in Table 3 highlighted medium–high Tocopherol content, with a fair variability among oil samples with values ranging from 402 ± 48 to 179 ± 21 mg kg^−1^; however, significant differences (*p* < 0.05) were depicted only in the Leccino and Lecciana varieties considering the two-harvest year. As previously observed for TPC and the antioxidant activity, Lecciana (B12), Frantene (B3) and the mix of L/D/P (C1) showed quite high content, with 402 ± 48, 349 ± 42 and 325 ± 39 mg kg^−1^, respectively. Similar results reported for both A/L oil samples (B4, C2), with values of 245 ± 29 and 280 ± 34 mg kg^−1^, were observed for Arbequina cultivar grown in SHD orchards, located in Spain [22]. As far as Koroneiki oil samples are concerned, our results (214.50 ± 27.8 mg kg^−1^) reflect the values recorded for the same cultivar grown in Greece [27]. Results of a two-way ANOVA (Table S1) highlighted that the harvest year did not affect, as a single factor, the TT content (*p* > 0.05) which is conversely significantly influenced by cultivar and the year/cultivar combination (*p* < 0.0001) as well as agronomic conditions, as reported by other authors for monovarietal oils [34].

### 3.3. Phenolic Profile

Due to the great variety of factors stated in the literature [35], including genetic, pedo-climatic, geographical origin, agronomic and technological factors, influencing the chemical composition of EVO, the comparison of phenolic profile and concentrations reported by different studies results quite difficult [36].

The characterization of phenolic profile of oil samples (Table 3) highlighted seven representative compounds belonging to three different classes: phenolic alcohol (hydroxytyrosol, tyrosol), flavonoids (diosmetin, luteolin, apigenin) and secoiridoid (oleacein and oleocanthal) (Table 3). Hydroxytyrosol (3,4-DHPEA), the most representative phenol in virgin olive oil, was found at levels below 6.5 mg L^−1^ in all olive oils, since Koroneiki (C4), Frantoio (C6) and Lecciana (C5) cultivars reported 6.3 ± 0.8, 5.9 ± 0.7 and 5.2 ± 0.6 mg L^−1^ values, respectively, similarly to those provided by previous authors for the same variety [5,36]. Most of cultivars showed good level of tyrosol (*p*-DHPEA), since content ranges from 6.3 ± 0.8 to 1.3 ± 0.2 mg L^−1^, associated with C6 and C2, respectively, similarly to those obtained for Spanish cultivars cultivated either in Spain and Morocco [35,36].

Concerning flavonoids compounds the allochthonous varieties showed higher content than the local cultivars. Apigenin is dictated as the substrate for the formation of luteolin in the flavonoid’s pathway by activity of a hydroxylase enzyme, and diosmetin is known to be a methoxy derivative of luteolin [36]. Sample B4 (Arbequina/Lecciana) showed the highest value of diosmetin 3.5 ± 0.4 mg L^−1^, similarly to that reported for Arbequina cultivated in Spain [36]. Luteolin and apigenin resulted in being higher in Lecciana, C5 and B12, with values of 8.1 ± 0.9 mg L^−1^ and 2.0 ± 0.2 mg L^−1^, respectively; all the oil samples showed values according to other authors [35,36].

Oleuropein is the predominant phenolic compound of the olive fruit and leaves of *Olea europaea* L. and does not occur in olive oil since the predominant phenolic compounds in the crushed paste and in the virgin olive oil are secoiridoid derivates, oleacein (dialdehyde form of decarboxymethyl elenolic acid linked to hydroxytyrosol; 3,4-DHPEA-EDA) [37] and oleochantal (*p*-HPEA-EDA), the aldehydic form of ligstroside. The high concentration of oleacein in olive oil is due to the hydrolysis of oleuropein catalyzed by endogenous glycosidases during the crushing of olive fruit [38] and its content broadly ranges from 111 to 285 mg kg^−1^ [39]. Frantene (B3) showed the highest value 349.9 ± 42.0 mg L^−1^, Koroneiki (B13) and L/D/P (B14) reported values within the aforementioned range (228.7 ± 27.4 and 168.7 ± 20.2 mg L^−1^) while the remaining samples reported the lower values (from 102.2 ± 12.3 to 33.4 ± 4.0 mg L^−1^).

Additionally, oleochantal, responsible for the burning pungent sensation of virgin olive oil [40], is generated from the malaxation step when precursors are exposed to enzymes; moreover, it has intense anti-inflammatory effects comparable to ibuprofen thanks to its capability to inhibit cyclooxygenases COX-1 and COX-2 but not 15-lipooxygenase [41] and therefore, this compound characterizes the nutraceutical properties of virgin olive oils. Our results highlighted high content of oleocanthal both in local and non-local varieties; mix of L/D/P (B14), Koroneiki (B13) and A/L (B4) reported 329.6 ± 39.6, 351.9 ± 42.2 and 256.1 ± 30.7 mg L^−1^, respectively, while the other varieties showed a medium/low level of such compound, similarly to those reported for Arbequina and Picual [42] and for Leccino cultivated in China [43]. Despite the different analytical technologies used to assess the phenolic profile, results of oleacein and oleocanthal in Koroneiki (B13) were in line with few results reported in literature for the same cultivar [44,45]. 

The results of one-way and two-way ANOVA confirmed the great influence of different genetic and agronomical factors in determining the phenolic profile of olive oils (Table S1).

### 3.4. Fatty acid Profile

Fatty acids represent the major olive oil compounds fraction and are strictly related to the different vegetable oils species. Due to their impact on oils nutritional value and oxidative stability, their characterization is an important quality parameter and authenticity indicator of virgin olive oils [2]. The analysis revealed (Table 4) that the content of some individual fatty acids, markers of genuineness, such as myristic, linolenic, arachidonic, eicosanoid, behenic and lignoceric acid were found within the limits defined by the official normal standard [13,14], while there were some differences in the remaining fatty acids composition associated with cultivar. 

Oleic acid concentration ranged from 70.3% to 78.3%, for Frantoio (B2) and Koroneiki (C4), respectively. The biennial values for Lecciana resulted in being significantly different (*p* < 0.05), 71.6% (B12) and 76.7% (C5); however, considering the mean values (data not shown), the result of this was in accordance with those observed for the same variety cultivated in the Puglia region [10]; similar results were observed for the mix of Arbosana/Lecciana (B4, C2) with respect to Lecciana monocultivar, regardless of the different growing area. Koreneiki registered lower oleic acid value in 2020 (73.2%) than 2021 (78.3%) with contents like those found in Greece (76%) [27] and California (77.7%) [5]. The three local varieties (Frantoio, L/D/P and Leccino) showed the lowest oleic acid content ranging from 70.3% to 73.7%, contrary to the high data registered in Umbria region with values of 76.4 and 75.6% [6], probably associated with the influence of different pedoclimatic conditions as frequently reported in the literature [6,22,46]. Indeed, observing both of the ANOVA results (Table 4 and Table S1), the year, cultivar and their combination affected the oleic acid value.

In general, there is a negative correlation between oleic and linoleic content in virgin olive oil since to a lower content of oleic acid corresponds to a higher amount of linoleic acid [5]. Indeed, considering the mean values of linoleic acid between the two crop seasons (data not shown), higher values were observed in L/D/P, Frantoio and Leccino with 9.1, 8.5 and 7.7%, respectively; on the contrary, Koroneiki, Lecciana and Frantene showed the lowest linoleic acid content (7.6, 7.4, and 6.5%, respectively). The correlation with climatic condition, reported by different authors, indicating an increase in linoleic acid content in hot/warm environmental conditions due to an increase in oleate desaturase activity [5,47] is not evident in our study since the temperatures of the sites are similar (Table 1 and Figure S1); however, it could not be excluded that the activity of microsomal oleate desaturase (FAD2), expressed by FAD2-2 gene, strictly correlated to the cultivar parameter, which influences the oleic and linoleic acid content [48].

Palmitic acid represents the main saturated fatty acid and the second major fatty acid recorded in all olive oils (Table 4). Analyzing palmitic acid values, high content was reported in the local varieties Frantoio, Leccino and L/D/P, with values ranging from 13.4 to 15.9%, thus greater than those reported for Leccino and Frantoio cultivated in the Umbria region [6]. On the contrary, the allochthonous varieties Lecciana, Frantene, Koroneiki and A/L, cultivated in SHD orchard, showed low values of palmitic acid ranging from 13.7 to 10%. The results corroborate the hypothesis that fatty acids are strictly correlated to the gene expression such as, in this case, the stearoyl-acyl carrier protein desaturase gene (SAD); indeed, TT-SAD.1 genotype was found to be associated with a higher proportion of monounsaturated fatty acids, mainly oleic acid, as well as with lower proportions of palmitic acid [49], as previously discussed for the allochthonous variety, rich in such fatty acid; furthermore, training systems can also affect the fatty acid composition; it was observed that fruit orientation and lightening can change the fatty acids synthesis [50]; indeed, the reduction in the photosynthesis, due to poor lighting, mainly associated with higher volume canopy characteristics of traditional systems, delays the fruit ripening resulting in lower oil content, higher oleic acid, and lower levels in palmitic and linoleic acids [51]. Since photosynthesis is supported by lightened leaves, pruning and orchard density could be relevant factors for olive oil composition [52]. These different outcomes suggested a synergistic effect of genetic and environmental factors on the fatty acid composition and temperature and light play a role in modulating oleic acid content and the related ratios [49].

### 3.5. Cluster Analysis

**Figure 2:**
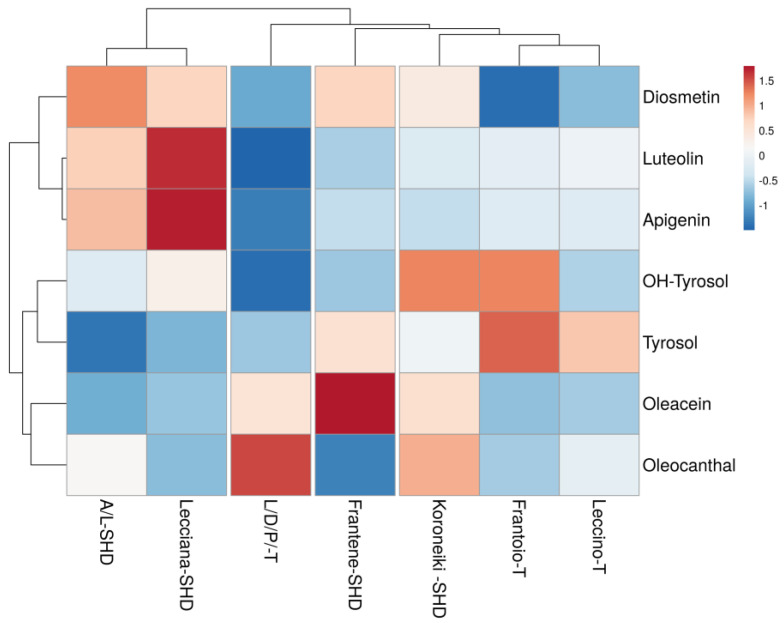
Phenolic compounds cluster heat map.

**Figure 3:**
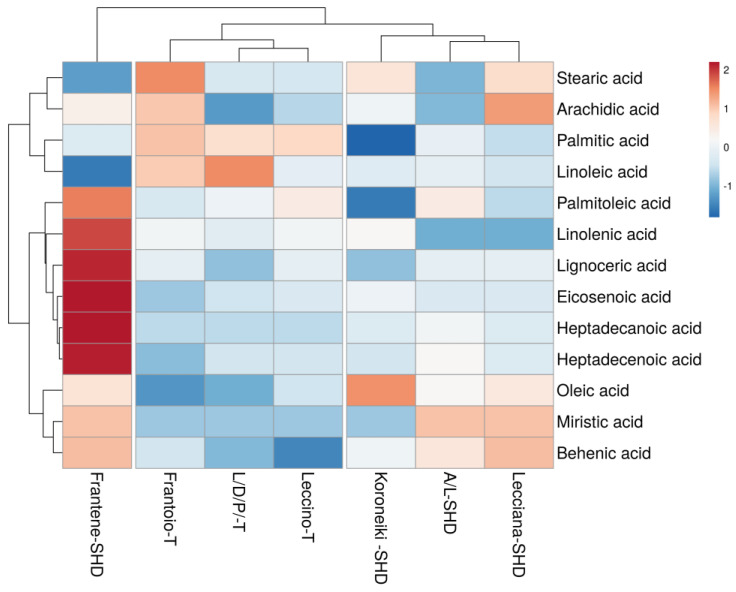
Fatty acids cluster heat map.

**Figure 4:**
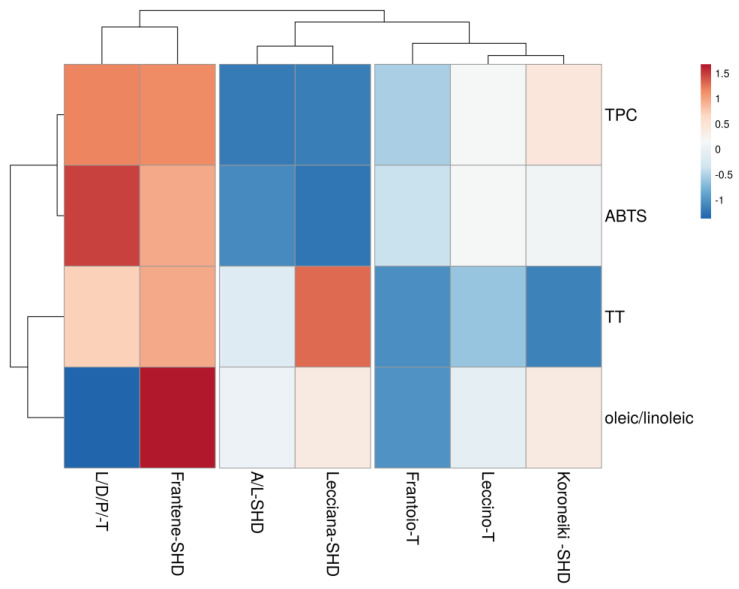
Main functional parameters cluster heat map.

To find similarities among the samples, depending on the varieties, growing system and site specifics, the cluster analysis of the biennial mean values was carried out, and a heatmap was produced (Figure 2, Figure 3 and Figure 4). 

The first dataset explored the functional parameters, in particular the phenolic profile. The results (Figure 2) showed how the cluster of Lecciana and mix of Lecciana/Arbequina grouped together mainly by the high content of flavonoids (diosmetin, luteolin and apigenin); a second cluster combined the local varieties Frantoio, Leccino and Koroneiki for the content of phenolic alcohols (tyrosol and hydroxytyrosol). Frantene and L/D/P can be considered separately because of their peculiar content of oleacin and oleocantal, respectively. 

The second cluster analysis was carried out with fatty acids data. Two main clusters can be observed (Figure 3). The first cluster, with all the local varieties, namely, Leccino, Frantoio and L/D/P, growing with traditional systems, had similarity mainly for high palmitic and linoleic acid content; the second cluster, with the allochthonous varieties cultivated in SHD, namely, Lecciana, Koroneiki and A/L, had similarity for oleic, miristic and beenic acid. Frantene was an outlier due to the high content of heptadecanoic, heptadecenoic, eicosenoic, lignoceric, linolenic and palmitoleic acid, with a good content of oleic, miristic and beenic acid, as observed for the other allochthonous variety. 

The last dataset combined quality parameters useful for a first quality index evaluation of the oil samples mainly related to health-promoting aspects. Four main functional parameters were supervised such as total phenolic content, antioxidant activity, total tocopherol content and oleic/linoleic ratio. Interesting and unexpected results (Figure 4) revealed some similarity between the mix of the local variety (Leccino/Dritta/Pendolino) and Frantene, despite the different cultivation area and the growing system. Despite the dissimilarity in the oleic/linoleic ratio, as discussed in Section 3.5, similarities in terms of TPC, TT and ABTS were observed.

## 4. Conclusions

The study conducted in the Abruzzo region highlights the good performance of the allochthonous olive cultivars Koroneiki, Arbequina, Frantene and Lecciana cultivated in super high-density orchards. In particular, the resulting related olive oils were rich in flavonoids and secoiridoids nutraceutical compounds. Oleic and linoleic fatty acid composition seem to be affected by the cultivar and the combined effect of the cultivar and year. Regarding the fatty acid profile, Frantene showed a peculiar pattern, one which was different from the other allochthonous cultivars grown in the same conditions. An interesting outcome is related to the similarity of oils from the mix of local variety (L/D/P) and Frantene in terms of health-related properties, confirming that despite the variability in pedoclimatic and agronomic conditions as well as the cultivar, it is possible to obtain high-quality olive oils comparable to traditional ones. The study could improve the olive oil sector, identifying olive varieties suitable for super high-density orchards and the potential development of super-sustainable olive groves, both in national and regional areas.

## Figures and Tables

**Table 1:** Orchard type, variety, climatic and orographic data of olive oil sample identified.

Orchard Type	Variety	Year	Sample Code	Location	Altitude (m)	DfS (km)	Average T (°C)	Rainfall (mm)
Traditional	Frantoio	2020	B2	Loreto Aprutino	170	18	15.8	620
		2021	C6				15.8	412
	L/D/P	2020	B14	Spoltore	80	6	16.2	440
		2021	C1				16.4	343
	Leccino	2020	B10	Pianella	169	15	16.6	563
		2021	C11				16.3	423
Super High-density	Frantene	2020	B3	Vasto	105	2	17.0	406
		2021	C13				16.7	515
	A/L	2020	B4	Casoli	104	20	15.7	395
		2021	C2				15.6	275
	Lecciana	2020	B12	Scerni	209	10	17.6	499
		2021	C5				17.4	434
	Koroneiki	2020	B13	Scerni	209	10	17.6	499
		2021	C4				17.4	434

L/D/P = Leccino/Dritta/Pendolino blend; A/L = Arbequina/Lecciana blend; DfS = distance from sea.

**Table 2:** Free Acidity, Peroxide Value, K232 and K270 of virgin olive oil samples.

Sample	Variety	Year	FA (Oleic Acid %)	PV (mEq O_2_/kg)	K_232_	K_270_
B2	Frantoio	2020	0.17 ± 0.02 ^c^	4.5 ± 0.54 ^bc^	1.68 ± 0.17 ^ab^	0.04 ± 0.01 ^c^
C6		2021	0.34 ± 0.04 ^a^	4.5 ± 0.54 ^bc^	1.40 ± 0.14 ^abc^	0.10 ± 0.01 ^ab^
B14	L/D/P	2020	0.20 ± 0.02 ^bc^	8.0 ± 0.96 ^a^	1.65 ± 0.17 ^abc^	0.05 ± 0.01 ^c^
C1		2021	0.34 ± 0.04 ^a^	5.0 ± 0.60 ^bc^	1.54 ± 0.15 ^abc^	0.10 ± 0.01 ^ab^
B10	Leccino	2020	0.34 ± 0.04 ^a^	4.0 ± 0.50 ^c^	1.36 ± 0.14 ^abc^	0.04 ± 0.01 ^c^
C11		2021	0.33 ± 0.04 ^a^	4.7 ± 0.56 ^bc^	1.37 ± 0.14 ^abc^	0.04 ± 0.01 ^c^
B3	Frantene	2020	0.35 ± 0.04 ^a^	5.0 ± 0.6 ^bc^	1.52 ± 0.15 ^abc^	0.04 ± 0.01 ^c^
C13		2021	0.15 ± 0.02 ^c^	5.0 ± 0.6 ^bc^	1.25 ± 0.13 ^bc^	0.10 ± 0.01 ^b^
B4	A/L	2020	0.17 ± 0.02 ^c^	5.0 ± 0.5 ^bc^	1.54 ± 0.15 ^abc^	0.04 ± 0.01 ^c^
C2		2021	0.19 ± 0.02 ^c^	4.2 ± 0.60 ^c^	1.23 ± 0.12 ^c^	0.12 ± 0.01 ^a^
B12	Lecciana	2020	0.35 ± 0.04 ^a^	4.0 ± 0.48 ^c^	1.72 ± 0.17 ^a^	0.04 ± 0.01 ^c^
C5		2021	0.13 ± 0.02 ^c^	4.0 ± 0.48 ^c^	1.34 ± 0.13 ^abc^	0.10 ± 0.01 ^ab^
B13	Koroneiki	2020	0.28 ± 0.03 ^ab^	4.5 ± 0.54 ^bc^	1.52 ± 0.15 ^abc^	0.05 ± 0.01 ^c^
C4		2021	0.37 ± 0.04 ^a^	6.0 ± 0.72 ^b^	1.24 ± 0.12 ^c^	0.04 ± 0.01 ^c^

Different lowercase letters, in the same column indicate significant differences (*p* < 0.05).

**Table 3:** Concentration (mg L^−1^) of individual phenols, (mg HTR kg^−1^) Total Phenolic Content (TPC) and antiradical activity (ABTS), (mg kg^−1^) Total Tocopherol (TT) in virgin olive oils.

Sample	Variety	Year	OH-Tyrosol	Tyrosol	Diosmetin	Luteolin	Apigenin	Oleacein	Oleocanthal	TPC	ABTS	TT
B2	Frantoio	2020	2.0 ± 0.2 ^bcd^	6.2 ± 0.7 ^ab^	0.9 ± 0.1 ^ef^	3.1 ± 0.4 ^cdef^	1.1 ± 0.1 ^c^	80.8 ± 9.7 ^de^	170.6 ± 20.5 ^cd^	310 ± 37 ^bcd^	420 ± 50 ^abc^	208 ± 25 ^def^
C6		2021	5.9 ± 0.7 ^a^	6.3 ± 0.8 ^a^	0.6 ± 0.1 ^f^	3.3 ± 0.4 ^bcde^	0.6 ± 0.7 ^defg^	51.5 ± 6.2 ^e^	76.8 ± 9.2 ^fgh^	140 ± 17 ^f^	220 ± 26 ^de^	228 ± 27 ^cdef^
B14	L/D/P	2020	0.7 ± 0.1 ^ef^	5.1 ± 0.6 ^abc^	1.4 ± 0.2 ^de^	1.0 ± 0.1 ^g^	0.5 ± 0.1 ^efg^	168.7 ± 20.2 ^c^	329.6 ± 39.6 ^a^	320 ± 38 ^bc^	512 ± 61 ^ab^	285 ± 34 ^bcde^
C1		2021	1.1 ± 0.1 ^def^	2.4 ± 0.3 ^ef^	0.7 ± 0.1 ^f^	1.9 ± 0.2 ^fg^	0.3 ± 0.0 ^g^	101.6 ± 12.2 ^d^	141.9 ± 17.0 ^cdef^	400 ± 48 ^ab^	520 ± 62 ^ab^	325 ± 39 ^abc^
B10	Leccino	2020	1.3 ± 0.1 ^def^	4.6 ± 0.6 ^bcd^	1.4 ± 0.2 ^de^	2.7 ± 0.3 ^def^	1.0 ± 0.1 ^c^	74.9 ± 9.0 d^e^	198.4 ± 23.8 ^bc^	286 ± 34 ^cde^	400 ± 48 ^abc^	179 ± 21 ^f^
C11		2021	2.6 ± 0.3 ^bc^	6.4 ± 0.8 ^a^	0.9 ± 0.1 ^ef^	4.1 ± 0.5 ^bc^	0.6 ± 0.1 ^defg^	70.2 ± 8.4 ^de^	103.9 ± 12.5 ^efg^	270 ± 32 ^cde^	350 ± 42 ^cd^	300 ± 36 ^bcd^
B3	Frantene	2020	0.7 ± 0.1 ^ef^	5.0 ± 0.6 ^abc^	2.6 ± 0.3 ^b^	2.3 ± 0.3 ^efg^	0.9 ± 0.1 ^cd^	349.9 ± 42.0 ^a^	152.7 ± 18.3 ^cde^	267 ± 32 ^cde^	390 ± 47 ^bc^	349 ± 42 ^ab^
C13		2021	2.9 ± 0.4 ^b^	5.4 ± 0.7 ^abc^	1.6 ± 0.2 ^cd^	2.9 ± 0.4 ^cdef^	0.5 ± 0.1 ^fg^	63.5 ± 7.6 ^de^	28.8 ± 3.5 ^h^	450 ± 54 ^a^	530 ± 64 ^a^	285 ± 34 ^bcde^
B4	A/L	2020	3.1 ± 0.4 ^b^	4.8 ± 0.8 ^abcd^	3.5 ± 0.4 ^a^	4.5 ± 0.5 ^b^	1.5 ± 0.2 ^b^	79.9 ± 9.6 ^de^	256.1 ± 30.7 ^b^	145 ± 17 ^f^	250 ± 30 ^de^	245 ± 29 ^cdef^
C2		2021	1.4 ± 0.2 ^def^	1.3 ± 0.2 ^f^	1.2 ± 0.2 ^def^	4.2 ± 0.5 ^bc^	0.8 ± 0.1 ^cde^	34.1 ± 4.1 ^e^	72.5 ± 8.7 ^gh^	200 ± 24 ^ef^	240 ± 29 ^de^	280 ± 34 ^bcde^
B12	Lecciana	2020	0.5 ± 0.1 ^f^	3.2 ± 0.4 ^de^	1.7 ± 0.2 ^cd^	3.1 ± 0.4 ^cdef^	2.0 ± 0.2 ^a^	33.4 ± 4.0 ^e^	109.5 ± 13.1 ^defg^	136 ± 16 ^f^	230 ± 28 ^de^	402 ± 48 ^a^
C5		2021	5.2 ± 0.6 ^a^	4.0 ± 0.5 ^cde^	2.5 ± 0.3 ^b^	8.1 ± 0.9 ^a^	1.1 ± 0.1 ^c^	102.2 ± 12.3 ^d^	123.0 ± 14.8 ^defg^	210 ± 25 ^def^	230 ± 28 ^de^	266 ± 32 ^bcdef^
B13	Koroneiki	2020	1.7 ± 0.2 ^cde^	4.9 ± 0.6 ^abc^	2.1 ± 0.3 ^bc^	2.2 ± 0.3 ^efg^	0.6 ± 0.1 ^defg^	228.7 ± 27.4 ^b^	351.9 ± 42.2 ^a^	448 ± 54 ^a^	520 ± 62 ^ab^	190 ± 23 ^ef^
C4		2021	6.3 ± 0.8 ^a^	4.3 ± 0.5 ^cd^	1.6 ± 0.2 ^cd^	4.0 ± 0.5 ^bcd^	0.7 ± 0.1 ^def^	52.2 ± 6.3 ^e^	61.6 ± 7.4 ^gh^	154 ± 18 ^f^	212 ± 25 ^e^	239 ± 29 ^cdef^

Different lowercase letters, in the same column indicate significant differences (*p* < 0.05).

**Table 4:** Fatty acid composition (%) of olive oils.

	B2	C6	B14	C1	B10	C11	B3	C13	B4	C2	B12	C5	B13	C4
	Frantoio		L/D/P		Leccino		Frantene		A/L		Lecciana		Koroneiki	
	2020	2021	2020	2021	2020	2021	2020	2021	2020	2021	2020	2021	2020	2021
Miristic acid (C14:0)	0.01 ^b^	0.03 ^ab^	0.01 ^b^	0.02 ^ab^	0.01 ^b^	0.03 ^ab^	0.01 ^b^	0.04 a	0.01 ^b^	0.04 ^a^	0.04 ^a^	0.02 ^ab^	0.01 ^b^	0.02 ^ab^
Palmitic acid (C16:0)	14.99 ^b^	14.70 ^b^	14.18 ^c^	14.77 ^b^	15.89 ^a^	13.40 ^ef^	13.56 ^de^	13.07 f	13.77 ^d^	13.37 ^ef^	13.35 ^ef^	12.64 ^g^	13.07 ^f^	9.91 ^h^
Palmitoleic acid (C16:1)	0.99 ^ef^	1.09 ^cde^	1.10 ^cde^	1.10 ^cde^	1.17 ^bcd^	1.19 ^bc^	1.36 ^a^	1.40 ^a^	1.23 ^b^	1.12 ^bcd^	0.93 ^f^	1.07 ^de^	0.92 ^f^	0.72 ^g^
Heptadecanoic acid (C17:0)	0.04 ^e^	0.05 ^de^	0.05 ^de^	0.05 ^de^	0.05 ^de^	0.04 ^e^	0.14 ^a^	0.11 ^b^	0.08 ^c^	0.06 ^cde^	0.06 ^cde^	0.05 ^de^	0.07 ^cd^	0.05 ^de^
Heptadecenoic acid (C17:1)	0.07 ^ef^	0.05 ^f^	0.09 ^de^	0.08 ^de^	0.09 ^de^	0.08 ^de^	0.27 ^a^	0.23 ^b^	0.16 ^c^	0.10 ^d^	0.10 ^d^	0.10 ^d^	0.09 ^de^	0.09 ^de^
Stearic acid (C18:0)	2.81 ^abc^	3.01 ^a^	2.39 ^ef^	2.48 ^def^	2.28 ^f^	2.57 ^cde^	2.48 ^def^	1.93 ^g^	2.26 ^f^	2.28 ^f^	2.98 ^ab^	2.48 ^def^	2.66 ^cd^	2.72 ^bcd^
Oleic acid (C18:1)	70.30 ^g^	71.76 ^f^	71.79 ^f^	71.12 ^fg^	71.31 ^fg^	73.69 ^d^	73.67 ^d^	75.09 ^c^	71.97 ^ef^	75.41 ^c^	71.62 ^f^	76.77 ^b^	73.18 ^de^	78.33 ^a^
Linoleic acid (C18:2)	9.25 ^ab^	7.96 ^e^	9.04 ^c^	9.06 ^bc^	7.72 ^f^	7.66 ^f^	6.90 ^g^	6.13 ^i^	9.18 ^bc^	6.28 ^i^	9.45 ^a^	5.50 ^j^	8.56 ^d^	6.67 ^h^
Linolenic acid (C18:3)	0.72 ^bc^	0.62 ^cde^	0.64 ^cde^	0.65 ^bcde^	0.71 ^bc^	0.63 ^cde^	0.86 ^a^	0.76 ^ab^	0.59 ^def^	0.56 ^ef^	0.65 ^bcde^	0.50 ^f^	0.70 ^bcd^	0.65 ^bcde^
Arachidic acid (C20:0)	0.43 ^a^	0.43 ^a^	0.35 ^a^	0.36 ^a^	0.37 ^a^	0.39 ^a^	0.37 ^a^	0.45 ^a^	0.36 ^a^	0.38 ^a^	0.44 ^a^	0.44 ^a^	0.37 ^a^	0.43 ^a^
Eicosenoic acid (C20:1)	0.23 ^b^	0.19 ^b^	0.24 ^b^	0.21 ^b^	0.25 ^b^	0.23 ^b^	0.23 ^b^	0.56 ^a^	0.24 ^b^	0.24 ^b^	0.23 ^b^	0.25 ^b^	0.25 ^b^	0.26 ^b^
Behenic acid (C22:0)	0.11 ^abc^	0.09 ^abc^	0.09 ^abc^	0.08 ^bc^	0.10 ^abc^	0.06 ^c^	0.11 ^abc^	0.14 ^a^	0.11 ^abc^	0.13 ^ab^	0.12 ^ab^	0.13 ^ab^	0.08 ^bc^	0.13 ^ab^
Lignoceric acid (C24:0)	0.05 ^ab^	0.02 ^b^	0.03 ^b^	0.02 ^b^	0.05 ^ab^	0.03 ^b^	0.04 ^ab^	0.09 ^a^	0.04 ^ab^	0.03 ^b^	0.03 ^b^	0.05 ^ab^	0.04 ^ab^	0.02 ^b^
Oleic/Linoleic	7.6 ^k^	9.0 ^h^	7.9 ^j^	7.9 ^j^	9.2 ^g^	9.6 ^f^	10.7 ^e^	12.3 ^b^	7.8 ^j^	12.0 ^c^	7.6 ^k^	14.0 ^a^	8.5 ^i^	11.7 ^d^
∑SFA	18.4 ^ab^	18.3 ^ab^	17.1 ^cd^	17.8 ^bc^	18.8 ^a^	15.5 ^de^	16.7 ^de^	15.8 ^e^	16.6 ^de^	16.3 ^de^	17.0 ^cd^	15.8 ^e^	16.3 ^de^	13.3 ^f^
∑MUFA	72.0 ^g^	73.1 ^ef^	73.2 ^ef^	72.5 ^fg^	72.8 ^fg^	75.2 ^d^	75.5 ^cd^	77.3 ^b^	73.6 ^ef^	76.9 ^bc^	72.9 ^fg^	78.2 ^ab^	74.4 ^de^	79.4 ^a^
∑PUFA	9.9 ^ab^	8.6 ^d^	9.7 ^b^	9.7 ^b^	8.4 ^d^	8.3 ^d^	7.8 ^e^	6.9 ^g^	9.8 ^b^	6.8 ^g^	10.1 ^a^	6.0 ^h^	9.3 ^c^	7.3 ^f^
MUFA/PUFA	7.2 ^i^	8.5 ^f^	7.6 ^h^	7.5 ^h^	8.6 ^f^	9.1 ^e^	9.7 ^d^	11.2 ^b^	7.5 ^h^	11.2 ^b^	7.2 ^i^	13.0 ^a^	8.0 ^g^	10.8 ^c^

Different lowercase letters, in the same column indicate significant differences (*p* < 0.05).

## Data Availability

Data available on request due to restrictions. The data presented in this study are available on request from the corresponding author.

## Supplementary Materials

The following supporting information can be downloaded at: https://www.mdpi.com/article/10.3390/foods12061292/s1, Figure S1: Mean thermo-pluviometry mereological data; Table S1: Two-way ANOVA results of qualitative characteristics of olive oil; Table S2: MS/MS parameters of the selected analytes for the MRM acquisition; m/z value (amu) of parent compounds in first quadrupole (Q1) and m/z values of the ion fragments in the third quadrupole (Q3).
