# Supplementary material for: The Introduction of Allochthonous Olive Variety and Super High-Density System in the Abruzzo Region: A Study on Olive Oil Quality
Source: Foods. 2023 Mar 17;12(6):1292. doi: 10.3390/foods12061292 (PMC10048017; doi:10.3390/foods12061292)
Supplement: Supplementary file 1 [file foods-12-01292-s001.zip › Table supplementary material.pdf]

Table S1. Two-way ANOVA results of qualitative characteristics of olive oil

	**Cultivar**	**Year**	**Cultivar*Year**
Free acidity	***	ns	***
Peroxides	***	ns	***
k_{232}	*	***	ns
k_{270}	***	***	***
Total phenolic compound	***	ns	***
Antiradical activity	***	**	***
Total Tocopherol	***	ns	***
OH-Tyrosol	***	***	***
Tyrosol	***	**	***
Diosmetin	***	***	***
Luteolin	***	***	***
Apigenin	***	***	***
Oleacein	***	***	***
Oleocanthal	***	***	***
Miristic acid (C14:0)	ns	***	*
Palmitic acid (C16:0)	***	***	***
Palmitoleic acid (C16:1)	***	ns	***
Heptadecanoic acid (C17:0)	***	**	*
Heptadecenoic acid (C17:1)	***	***	***
Stearic acid (C18:0)	***	ns	***
Oleic acid (C18:1)	***	***	***
Linoleic acid (C18:2)	***	***	***
Linolenic acid (C18:3)	***	***	**
Arachidic acid (C20:0)	**	**	ns
Eicosenoic acid (C20:1)	***	***	**
Behenic acid (C22:0)	**	ns	*

Lignoceric acid (C24:0)	*	ns	*
Oleic/Linoleic	***	***	***
∑SFA	***	***	***
∑MUFA	***	***	***
∑PUFA	***	***	***
MUFA/PUFA	***	***	***

ns: not significant; *:<0.05; **:<0.001; ***:<0.0001

Table S2. MS/MS parameters of the selected analytes for the MRM acquisition; m/z value (amu) of parent compounds in first quadrupole (Q1) and m/z values of the ion fragments in the third quadrupole (Q3).

**Compounds**	**Q1 (amu)**	**DP (V)**	**EP (V)**	**Q3 (amu)**	**CE (V)**	**CXP (V)**
OH-Tyrosol	152.9	−75.00	−7.00	122.9	−22.00	−8.00
				104.6	−30.00	−7.00
Tyrosol	137.0	−65.00	−9.00	119.0	−21.00	−8.50
				106.9	−23.00	−5.50
Luteolin	284.9	−100.00	−8.00	150.9	−35.00	−11.00
				199.0	−35.00	−6.00
Apigenin	268.9	−110.00	−4.00	116.9	−50.00	−9.00
				151.0	−34.00	−5.00
Diosmetin	299.1	−90.00	−6.00	255.8	−40.00	−9.00
				150.9	−40.00	−10.00

**Compounds**	**Q1 (amu)**	**DP (V)**	**EP (V)**	**Q3 (amu)**	**CE (V)**	**CXP (V)**
Oleacein	318.9	−50.00	−10.00	195.0	####	−7.00
				183.0	####	−6.00
Oleocanthal	303.1	−40.00	−5.00	285.1	####	−9.00
				179.1	####	−6.00
