# Supplementary figures and images for: The Introduction of Allochthonous Olive Variety and Super High-Density System in the Abruzzo Region: A Study on Olive Oil Quality
Source: Foods. 2023 Mar 17;12(6):1292. doi: 10.3390/foods12061292 (PMC10048017; doi:10.3390/foods12061292)

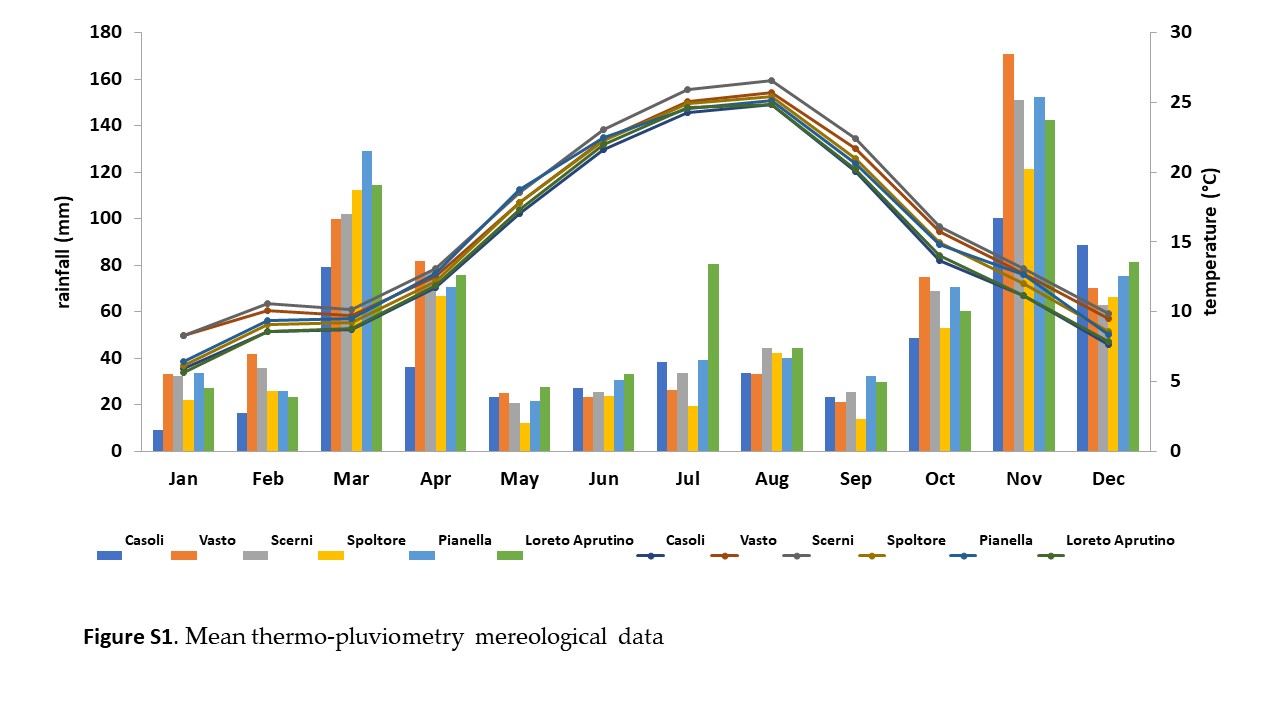

Supplement: Supplementary file 1 [file foods-12-01292-s001.zip › FS1.jpg]
